# Coatings of Different Carbon Nanotubes on Platinum Electrodes for Neuronal Devices: Preparation, Cytocompatibility and Interaction with Spiral Ganglion Cells

**DOI:** 10.1371/journal.pone.0158571

**Published:** 2016-07-06

**Authors:** Niklas Burblies, Jennifer Schulze, Hans-Christoph Schwarz, Katharina Kranz, Damian Motz, Carla Vogt, Thomas Lenarz, Athanasia Warnecke, Peter Behrens

**Affiliations:** 1 Institute for Inorganic Chemistry, Leibniz University Hannover, Hanover, Germany; 2 Department of Otorhinolaryngology, Head and Neck Surgery, Hannover Medical School, Hanover, Germany; 3 Cluster of Excellence Hearing4all, Hanover, Germany; University of Antwerp, BELGIUM

## Abstract

Cochlear and deep brain implants are prominent examples for neuronal prostheses with clinical relevance. Current research focuses on the improvement of the long-term functionality and the size reduction of neural interface electrodes. A promising approach is the application of carbon nanotubes (CNTs), either as pure electrodes but especially as coating material for electrodes. The interaction of CNTs with neuronal cells has shown promising results in various studies, but these appear to depend on the specific type of neurons as well as on the kind of nanotubes. To evaluate a potential application of carbon nanotube coatings for cochlear electrodes, it is necessary to investigate the cytocompatibility of carbon nanotube coatings on platinum for the specific type of neuron in the inner ear, namely spiral ganglion neurons. In this study we have combined the chemical processing of as-delivered CNTs, the fabrication of coatings on platinum, and the characterization of the electrical properties of the coatings as well as a general cytocompatibility testing and the first cell culture investigations of CNTs with spiral ganglion neurons. By applying a modification process to three different as-received CNTs via a reflux treatment with nitric acid, long-term stable aqueous CNT dispersions free of dispersing agents were obtained. These were used to coat platinum substrates by an automated spray-coating process. These coatings enhance the electrical properties of platinum electrodes, decreasing the impedance values and raising the capacitances. Cell culture investigations of the different CNT coatings on platinum with NIH3T3 fibroblasts attest an overall good cytocompatibility of these coatings. For spiral ganglion neurons, this can also be observed but a desired positive effect of the CNTs on the neurons is absent. Furthermore, we found that the well-established DAPI staining assay does not function on the coatings prepared from single-wall nanotubes.

## Introduction

Neural interface electrodes have been successfully used in clinical applications, for instance in cochlear implants and deep brain stimulation [1]. Cochlear implants have been, for almost over 30 years now, the gold standard in the treatment of patients suffering from profound or complete sensorineural hearing loss [2]. In recent years, even patients with residual hearing have become candidates due to a relaxation of the cochlear implantation criteria. For patients with residual hearing in the lower frequencies, a combination of electric and acoustic stimulation in the same ear provides an approach for successful hearing restoration alongside with preservation of residual hearing [3]. Despite huge technological and clinical progress, there are still fundamental requirements related to the utilization of neural interface electrodes. Current research focuses on the increase of the long-term electrode functionality and the reduction of the size of the electrode contacts without losing the ability of effective charge transfer. For more effective, smaller and safer electrodes, material concepts have to be developed which–while respecting biocompatibility and chemical stability–provide high electrical conductivities and possibilities for implant-associated drug delivery [4]. Carbon nanotubes (CNTs) are a promising base material for these purposes. They feature high electrical conductivity and mechanical strength; without further modification they are chemically rather inert and electrochemically stable [5–8]. With appropriate surface modifications, an increase of the surface area of the electrode contacts can be achieved. Because of these remarkable properties, several research groups have presented carbon nanotube preparations for neural interface applications over the last decade for the following purposes: promotion of neurite outgrowth [9], enhancement of neuronal recording [10] or stimulation performance [11], provision for local drug delivery [12].

The interaction of CNTs with neuronal cells has been studied for different types of CNTs (single-wall, multi-wall) and for a variety of neurons. It is difficult to compare and interpret the results as they appear to depend strongly on the kind and preparation of the CNTs and on the type of neurons [9,13,14]. However, CNTs have been shown in some studies to serve as an extracellular matrix for neurons and to direct neurite outgrowth, regulate neurite branching as well as to provide adhesion points for neurons [15]. This makes CNTs a promising matrix for primary neuronal cell cultures [16] as an alternative to other established matrices (e.g. matrigel, laminin or poly-D/L-ornithine). Additionally, CNTs were able to influence the secretion of neuroprotective factors like brain-derived neurotrophic factor (BDNF) [16]. In the field of neuronal prostheses, CNT coatings could–apart from their excellent properties–provide a functionalization of the electrode surface which is favorable for neurons, possibly reducing foreign body reactions and immune response. The investigation of such coatings therefore appears promising.

The cochlear implant electrically stimulates spiral ganglion neurons (SGNs), the primary auditory neurons in the inner ear. To the best of our knowledge, the interaction of this specific type of neuron with CNTs has not been investigated so far. The electrode array of cochlear implants consists of several platinum contacts embedded in a silicone matrix. Correspondingly, we chose platinum as a substrate for the deposition of CNT films. The preparation of CNT-containing films is often carried out using additional ingredients, like dispersing agents [6,17] or polymer matrices [12,18] for the formation of composite films. In contrast, we preferred to apply coatings made from pure nanotube dispersions in order not to mask possible effects of the CNTs on the cells or to register effects related to the dispersing agents. We employed CNTs from three different sources, Baytube multi-wall CNTs as well as two types of single-wall CNT products. After applying a suitable purification procedure to the CNTs, the coatings were applied from stable suspensions by a simple, low-temperature spray-coating method. The automated spray-coating technique results in a high homogeneity of the coating and a very good reproducibility for large numbers of samples (as necessary for cell-culture studies) can be ensured. Whereas the set-up of our experiments is adapted for a potential future application in cochlear implants, the main purpose of this study is to test the cytocompatibility of different types of CNT coatings with SGNs.

## Materials and Methods

### Carbon nanotube purification and characterization

Single-walled CNTs from two different suppliers (SWeNT CG200, SouthWest NanoTechnologies, USA and SWCNT, Fraunhofer IWS Dresden, Germany) and multi-walled CNTs (Baytubes C 70 P, Bayer MaterialScience, Germany) were purified and carboxyl-modified via treatment in oxidizing acid [19,20]. Specifically, the as-received CNT preparations were refluxed for 6 h in nitric acid (70%, Sigma Aldrich, Germany). Several washing and centrifugation steps with ultrapure water were performed until the pH value of the CNT dispersions is above 2 and the CNTs are no longer separable by centrifugation. A dialysis for one week against ultrapure water followed (Carl Roth Visking, 14 kDa MWCO, Carl Roth, Germany for large volumes (250 mL) and Sigma Aldrich Pur-A-Lyzer Mega 600 Dialysis Kit, 6−8 kDa MWCO, Sigma Aldrich, Germany for small volumes (20 mL)). Subsequent freeze-drying led to a lightweight and voluminous powder of purified CNTs.

The different CNT materials were characterized prior to the acid treatment and afterwards using different methods. Scanning electron microscope (SEM) images were obtained on a Jeol JESM-6700F field emission SEM (JEOL, Germany) with a semi in-lens detector at a working distance of 3 mm and an acceleration voltage of 2 kV. Raman spectra were measured on a Bruker Senterra Raman microscope (Bruker, USA) with a laser wavelength of 532 nm and a laser power of 10 mW. Thermogravimetric (TG) measurements were performed with Netzsch STA 429 (Netzsch, Germany) under flowing air from 35°C to 1000°C with a heat rate of 5 K∙min^−1^. The data were analyzed with the software Proteus Thermal Analysis 4.3.1 (Netzsch, Germany).

### Carbon nanotube dispersions

The purified CNTs were dispersed in ultrapure water (mass fraction of CNTs: 0.1%) by pre-dispersing in an ultrasonic bath and subsequent high energy dispersing for 1 h with an ultrasonic disintegrator (160 W, Branson Digital Sonifier 450D, Branson Ultrasonics, USA). By this treatment, long-term stable (months to years) aqueous CNT dispersions are accessible. Dispersions utilizable for spray-coating were prepared by dilution with ultrapure water to CNT mass fractions of 0.01%.

For further characterization of the carbon nanotube preparations, inductively coupled plasma optical emission spectrometry (ICP-OES) analysis of the CNT dispersions were performed to obtain information of the elemental composition. A procedure based on the work of Yang *et al*. (2010) was used to extract the metal residues on and encapsulated within the carbon nanotubes via microwave-assisted treatment with nitric acid and hydrogen peroxide [21]. For all three different CNTs, 3 g of the dispersion were mixed with 3 ml nitric acid (65%, subboiled, Sigma Aldrich, Germany) and 4 mL hydrogen peroxide (30%, Sigma Aldrich, Germany) followed by a microwave digestion (turboWAVE, MLS GmbH, Germany). According to Yang *et al*. (2010), a two-step procedure was used. The temperature was ramped to 170°C in 20 min with 1200 W, followed by a dwelling time of 5 min, then ramped to 185°C with 1200 W, followed by a dwelling time of 15 min [21]. After cooling down to room temperature, the samples were diluted with ultrapure water to 30 g. Before analysis, all samples were filtered through a metal-free PTFE filter (0.45 μm, VWR, Germany). For ICP-OES analysis, a Spectro ARCOS system (Spectro Analytical Instruments GmbH, Germany) was used. Selection of the metals to be analyzed was based on a consensus between the three samples and the highest detected intensities, i.e. highest contents. The following metals were investigated and quantified in all samples (applied calibration ranges in brackets): cobalt (15−100 μg·kg^−1^), iron, molybdenum, nickel, zinc (20−100 μg·kg^−1^), magnesium (50−100 μg·kg^−1^) and calcium (50−2500 μg·kg^−1^). If necessary, further dilution of the sample was performed. Turbidimetric measurements of CNT dispersions in ultrapure water were performed in range of 300 to 800 nm with a UV-vis spectrometer (Varian Cary 5E, Agilent Technologies, USA) in a quartz cuvette.

### Platinum substrates

Silicon wafers (4 inch diameter, 525 μm thickness, Si orientation <100>, Siegert Wafer, Germany) were metalized after cleaning and pre-processing with a 50 nm titanium adhesion layer and a 100 nm platinum layer by the Institute of Micro Production Technology (IMPT, Leibniz University Hannover, Germany). The wafers were mechanically separated into squares (1.5 x 1.5 cm²) to be used as substrates for spray-coating and cell culture investigations.

### Spray-coating process

Prior to the coating process, the platinum substrates were treated with freshly prepared piranha solution (H_2_SO_4_, 98% and H_2_O_2_, 30% in ratio of 2:1) for 30 min. Thereafter, the samples were successively rinsed with ultrapure water, ethanol and isopropanol, followed by drying in a stream nitrogen. To preserve optimal wettability of the substrates, the coating process was performed immediately after the cleaning process.

In preparation for the spray-coating process 15 platinum substrates were fixed with an aluminum sample holder and heated up to 125°C. An automated spray-coating robot (Walther Systemtechnik GmbH, Germany) was used to coat all 15 samples simultaneously in one process to ensure equal conditions for each of the samples. Compressed nitrogen was used as carrier gas. The used vaporizing pressure was 1 bar. To achieve homogenous coatings, the nozzle spraying the suspensions was slightly opened and 500 spraying steps were performed, spraying only very small amounts of material within each step.

### Characterization of CNT films

The topography of the CNT films was examined via field emission scanning electron microscopy (SEM, Jeol JSM-6700F, JEOL, Germany, working distance 3 mm, acceleration voltage 2 kV) and confocal microscopy (Leica DCM 3D, Leica Microsystems, Germany, EPI 150X-L objective, 3 μm confocal z-scan). By mechanically removing part of the CNT coatings, determination of the film thicknesses was possible using confocal microscopy. Removal of a CNT coating without damaging the smooth platinum surface of the substrate was possible by applying with a plastic spatula strong contact pressure. Evaluation of the confocal microscope images was made with the Software LeicaSCAN DCM3D 3.2.3 (Leica Microsystems, Germany).

Survey X-ray photoelectron spectra of the CNT films were acquired on a Versa Probe spectrometer (Physical Electronics, USA) at a base pressure of less than 10^−8^ mbar using monochromatic Al K_α_ X-ray photons (1486.68 eV) irradiating at 45° relative to the electron analyzer entrance. The photo-electrons were analyzed by a concentric hemispherical analyzer operated at constant pass energy 187.5 eV for survey spectra. The photo-emission angle (*θ*) was set to 54.7°. The X-ray gun was operated at 50 W with a spot size of 200 μm. For each type of CNT coatings two samples at two different positions were investigated.

Non-coated substrates and spray-coated samples were investigated with regard to their electrical properties by potentiostatic electrochemical impedance spectroscopy (PEIS) in order to determine the influence of the CNT coating. The measurements were performed by using a potentiostat (Princeton Applied Research Versastat 4, Ametek, USA) and a standard three-electrode electrochemical cell configuration with a Pt wire as counter electrode and an Ag/AgCl reference electrode (saturated KCl-AgCl solution). Non-coated and spray-coated samples were electrically contacted with a Cu wire (1 mm diameter, Sigma Aldrich, Germany) and silver conductive paint. Saline solution (0.85% NaCl, Sigma Aldrich, Germany) was used as electrolyte and alternating current with an amplitude of 10 rms mV was applied. By masking with adhesive tape, the active electrode surface which was exposed to the solution was limited to an area of 1.5 x 1.0 cm². The values of the impedance were determined at ten frequencies per decade over the range form 10^−1^ to 10^5^ Hz. The data were analyzed with the software ZView Version 3.3e (Scribner Associates Inc., USA).

### Cell culture investigations on CNT-coated samples

#### NIH3T3 fibroblasts

The murine fibroblast cell line NIH3T3 lentivirally infected for the expression of green fluorescent protein (GFP) as a marker was used for the initial cytocompatibility tests. The generation of the lentiviral vector as well as the infection procedure were described in detail elsewhere [22]. For the experiments, the cells were used at passages three to six.

The NIH3T3 fibroblasts were cultured in Dulbecco’s Modified Eagle’s Medium (DMEM, high glucose; Biochrom, Germany) supplemented with 10% fetal calf serum (FCS; Biochrom, Germany) as well as 1% penicillin and streptomycin (Biochrom, Germany) at standard conditions (37°C, 5% CO_2_). Cells were seeded with a density of 1 x 10^4^ cells per sample in a 6-well plate (Greiner Bio-One, Germany). Allowing the cells to adhere to the samples for 24 h, they were thereafter transferred into a new 6-well plate. Daily, the morphology and proliferation of the fibroblasts were checked with a reflected light microscope (Olympus BX51, Olympus, Germany) and representative images were captured. After four days, the cells were detached from the samples with a trypsin/EDTA solution (0.25%; Biochrom, Germany) for quantification. The number of viable cells was determined in duplicates by using a Neubauer chamber (Neubauer improved, Brand, Germany) and the trypan blue exclusion test (dilution 1:2; Sigma-Aldrich, Germany).

#### Ethics statement for isolation of SGN from neonatal rats

The experiments and analysis of this study were conducted from March 2014 to October 2015. They were performed in accordance with the institutional guidelines for animal welfare of the Hannover Medical School following the standards described by the German ‘Law on Protecting Animals’ (Tierschutzgesetz) and with the European Directive 2010/63/EU for protection of animals used for experimental purposes. The euthanasia for our in vitro experiments is registered (no.: 2013/44) with the local authorities (Zentrales Tierlaboratorium, Laboratory Animal Science, Hannover Medical School, including an institutional animal care and use committee) and reported on a regular basis as demanded by law. For exclusive sacrifice of animals for tissue analysis in research, no further approval is needed if no other treatment is applied beforehand (§4). The rats were bred and born for research study purposes. A breeding stock was supplied by Charles River (Charles River, USA) and housed with their litters in the facilities of the licensed Institution of Laboratory Animal Science of the Hannover Medical School. To minimize the stress level for the neonatal rats, they were euthanized by decapitation prior to any experimentation by a licensed person.

#### Spiral ganglion cells

In order to investigate the action of the different types of CNT coatings on neuronal cells, freshly isolated spiral ganglion cells (SGCs) were cultivated on CNT-coated platinum samples. The primary cell culture of SGCs was prepared from neonatal Sprague-Dawley rats. For this purpose, the cochleae were isolated and microscopically dissected after decapitation of the animals. According to a previously described protocol [23], the enzymatic and mechanical dissociation of the spiral ganglia was performed. Afterwards, the number of viable cells was determined by using a Neubauer chamber (Neubauer improved, Brand, Germany) and the trypan blue exclusion test (dilution 1:2; Sigma-Aldrich, Germany). The platinum-based samples to be tested were placed in a 12-well culture plate (TPP Techno Plastic Products, Switzerland). The dissociated SGCs were seeded at a density of 4 x 10^4^ cells per sample per well. The SGC cultures were cultivated at standard conditions in SGC medium, i.e., serum-free medium (Panserin 401, PAN Biotech, Germany) supplemented with HEPES (25 mM final concentration; Life Technologies, USA), glucose (6 mg·mL^−1^; Braun AG, Germany), penicillin (30 U·mL^−1^; Grünenthal GmbH, Germany), N2-supplement (3 μg·mL^−1^; Life Technologies, USA) and insulin (5 μg·mL^−1^; Sigma-Aldrich, Germany). After an incubation time of 48 h, the SGCs were fixed with PFA (4%) for 10 min and washed with phosphate-buffered saline (PBS; PBS tablets, Gibco® by Life Technologies, USA). The SGCs were cultivated on three different CNT coatings (Bayer MWNTs, Fraunhofer SWNTs and SWeNT SWNTs). Uncoated platinum and cell culture wells coated with poly-D/L-ornithine (0.01 mg·mL^−1^; Sigma-Aldrich, USA) and laminin (0.1 mg·mL^−1^; natural mouse, Life Science Technologies, USA) served as control.

#### Immunofluorescence staining

The dissociated SGC cultures were mixed cultures containing neurons, fibroblasts and glial cells. For the identification of spiral ganglion neurons (SGNs) in the mixed SGC culture, the anti-neurofilament antibody (200 kDa, monoclonal mouse, clone RT97; Leica Biosystems, Germany) was used as a neuron-specific marker. The glial cells were stained with the anti-S100 antibody (S100 isoform, polyclonalrabbit, abcam, UK). All cells cultured on platinum samples were fixed with PFA (4%) and permeabilized with 0.25% Triton X-100 (Sigma-Aldrich, Germany) in PBS for 10 min. After three times of washing for 5 min with 0.1% Triton X-100 in PBS (PBT), the samples were incubated for 1 h in blocking buffer containing 5% FCS (Biochrom, Germany) diluted in PBT. Cells were washed three times for 3 min with PBT and were incubated with the primary antibodies anti-neurofilament (1:500) and anti-S100 (1:50) diluted in antibody dilution buffer containing 2% FCS (Biochrom, Germany) and 1% bovine serum albumin (BSA; Sigma-Aldrich, Germany) in PBT at 4°C over night. Afterwards, the samples were washed three times for 5 min with PBT and were incubated with the fluorescence-conjugated secondary antibodies Alexa Fluor^®^488 (1:1000, goat anti-mouse, Jackson ImmunoResearch, USA) and Alexa Fluor^®^594 (1:500, goat-anti rabbit, Jackson ImmunoResearch, USA) as well as 4′,6-Diamidin-2-phenylindol (DAPI; 1:250, AppliChem, Germany) for 1 h at room temperature. Finally, the samples were washed again and stored in PBS. Samples were imaged with a reflected light microscope (Olympus BX51, Olympus, Germany) and the number of survived SGNs was determined by counting all neurofilament positive cells with a neurite length of at least three cell soma diameters [24].

#### CNT supernatants

Supernatants of CNT-coated platinum samples were collected after an incubation of 5 days in DMEM, supplemented with 10% FCS (Biochrom, Germany) as well as 1% penicillin and streptomycin (Biochrom, Germany). In order to investigate the biocompatibility of CNT-coatings and to examine the possible release of components in the cell culture medium, the collected supernatants were added to freshly isolated SGCs (see above). The dissociated SGCs were seeded at a density of 1 x 10^4^ cells per well in a 96-well plate (Nunc, Nunclon Surface, Thermo Fisher Scientific, USA) and were cultured for 48 h in a (1:1) mixture of SGC medium and the collected supernatants of the different CNT-coated samples. A seeding control was fixed already 4 h after plating. As a control, SGCs were cultivated in serum-deprived SGC medium (medium control), in SGC medium supplemented with 50 ng/mL BDNF (Invitrogen by Life Technologies, USA) (BDNF control) as well as in serum-containing medium (FCS control). After an incubation time of 48 h, the cells were fixed with a 1:1 acetone (J. T. Baker, Netherlands) and methanol (Carl Roth, Germany) solution for 10 min and washed with phosphate-buffered saline (PBS; PBS tablets, Gibco® by Life Technologies, USA).

For the quantification of neuronal survival, a neuron-specific staining was used to identify the SGNs. Fixed cells were thus stained with a mouse 200 kD anti-neurofilament antibody (clone RT97; Leica Biosystems, Germany), a secondary biotinylated anti-mouse antibody and ABC complex solution using the Vectastain® Elite® ABC Kit (Vector Laboratories Inc., USA)–as described in detail in ref. [23]. The antibody complexes were visualized by adding diaminobenzidine (Peroxidase Substrate Kit DAB, Vector Laboratories Inc., USA). Surviving neurons were defined as neurofilament-positive cells exhibiting a neurite length of at least three cell soma diameters [24] and were counted by using an inverted microscope (Olympus CKX41, Olympus, Germany). The survival rate was determined by relating the number of survived neurons per well to the mean seeding density (mean number of neurons in the seeding control) of the same plate.

### Statistical analysis

Statistical analysis was performed with Prism 5 (GraphPad, USA). The results were validated by using one-way analysis of variance (ANOVA) followed by Bonferroni’s multiple comparison test. *P* values of less than 0.05 were considered to be statistically significant. All quantitative data represent the means of at least three independent approaches (*N*), including at least duplicates of each sample (*n*). Error bars in the figures indicate the standard error of the mean. Levels of significance are indicated as follows: *p < 0.05; **p < 0.01; ***p < 0.001.

## Results and Discussion

### Purified carbon nanotubes

**Fig 1:**
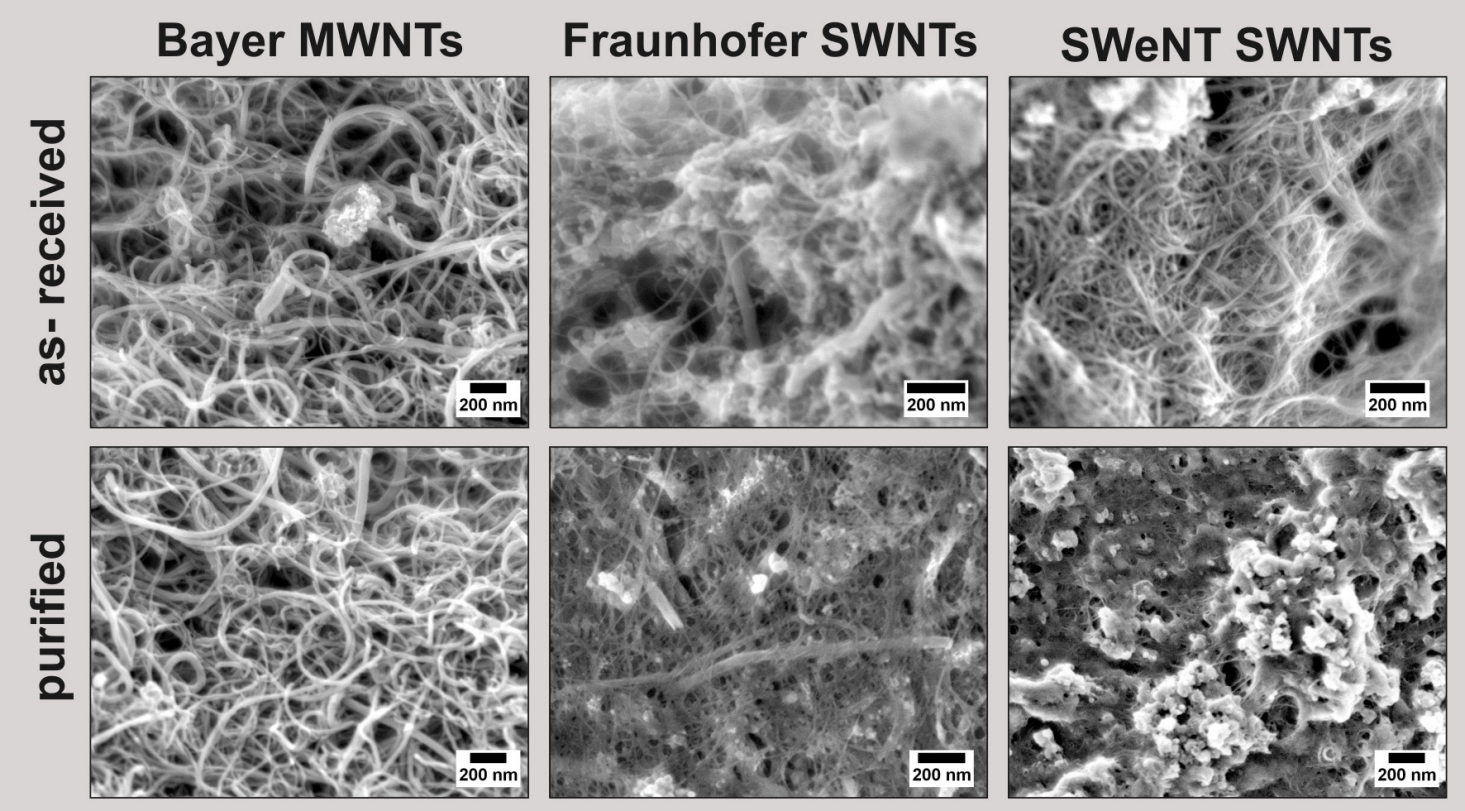
SEM images of as-received (*top*) and purified (*bottom*) CNTs: Bayer MWNTs, Fraunhofer SWNTs, SWeNT SWNTs (*left to right*).

Investigations with SEM provided information about the lengths and thicknesses of the used carbon nanotubes. According to the supplier's information the diameters of the single-wall nanotubes should be in the range of 1 nm and the length should be higher than 1 μm. Multi-wall nanotubes should have varying diameters of 5 to 20 nm and similar lengths. The SEM images confirm these data (Fig 1). Bayer MWNTs consist of a mixture of nanotubes with different diameters (Fig 1 left). Fraunhofer SWNTs show mainly very thin nanotubes but also a few with very large diameters (Fig 1 center); in contrast, the SWeNT SWNTs consistently exhibit small diameters (Fig 1 right). The images of the as-received CNT powders (Fig 1 top) reveal the presence of residual inorganic catalyst particles stemming from the fabrication process. The Fraunhofer SWNTs show the highest content of these particles in the SEM images (Fig 1 center top). After the purifying acid treatment, the quantity of catalyst particles is reduced for all the different types of purified CNTs (Fig 1 bottom). However, in case of the purified SWeNT SWNTs, deposits with another morphology than the residual catalyst particles are visible (Fig 1 bottom right).

The thermogravimetric analysis, carried out under flowing air, of the as-received and purified CNT powders is consistent with the information gathered from the SEM investigations. Table 1 lists the percentages of the residual masses after thermogravimetric analysis of as-received and purified CNT powders. The percentages correspond to oxides of the catalyst constituents. As expected, Fraunhofer SWNTs show the highest residual amount of inorganic components both before and after purification, but a reduction from 29 to 14 wt% was achieved by the acid treatment. Supplier information for the as-received Fraunhofer SWNTs declare ca. 70% SWNT content. The as-received Bayer MWNTs already exhibit a high purity of 97 wt% (supplier specification: >95%), nevertheless a further reduction of the inorganic content to 1 wt% was possible by the purification process. Only SWeNT SWNTs show a slight increase from 6 to 9 wt% for the residue, potentially due to the uptake of soluble inorganic salts incorporated during dialysis processes or to a partial removal of carbon materials due to oxidation. The shapes of the TG curves (depicted in S1 Fig) gave no further information on modification or purity of the CNTs.

**Table 1:** Residues of carbon nanotube preparations after thermogravimetric analysis under flowing air. The method has s typical error of ca. 1%.

CNT	type	residue / %	
		as-received	purified
Baytubes C70P	MWNT	3	1
Fraunhofer SWNT	SWNT	29	14
SWeNT CG200	SWNT	6	9

ICP-OES analysis of the CNTs provided information on the metal content of the dispersions (Table 2). With this analysis further statements regarding the composition of the inorganic residue of the dispersions are possible. Catalyst metals typically used in the preparation of CNTs, namely cobalt, iron, molybdenum and nickel, were found and determined; also the alkaline earth metals calcium and magnesium, as well as zinc, were detected in significant amounts. The total quantities of the metal content within the dispersions showed a quite good correlation with the amounts of inorganic residues of the CNT powders after thermogravimetric analysis. In total, the Fraunhofer SWNTs exhibited the highest metal content in the dispersion and the Bayer MWNTs the lowest. Transition metals showed very different contents for the three different CNT dispersions, obviously depending on the catalyst used by the manufacturer. Because of the small contents detected and with regard to the poor solubility of the residual catalyst particles and to their encapsulation within the CNTs, cytotoxic effects of these metals may be limited due to reduced bioavailability [21]. All three dispersions showed high contents of earth alkaline metals calcium and magnesium probably originating from catalyst supports.

**Table 2:** Metal contents of the different CNT dispersions determined via ICP-OES. Metal contents refer to the dispersions with CNT mass fractions of 0.1%. DL: detection limit.

CNT dispersion	metal content / μg·kg^-1^							
	Co	Fe	Mo	Ni	Zn	Mg	Ca	total
Bayer MWNTs	284.3 ± 18.7	266.6 ± 18.4	< DL	60.9 ± 21.5	262.59 ± 4.54	1959.1 ± 23.1	6575 ± 452	9408.49 ± 453.88
Fraunhofer SWNTs	15697 ± 380	4943 ± 126	668.8 ± 15.3	16956 ± 371	907.06 ± 4.49	734.4 ± 23.8	14350 ± 468	54256.26 ± 719.56
SWeNT SWNTs	181.9 ± 21.5	554.1 ± 17.4	2252.8 ± 62.8	132.1 ± 21.4	497.69 ± 4.32	6039.6 ± 89.8	25915 ± 471	35573.19 ± 484.86

Raman spectroscopy is a common method to investigate carbon nanomaterials, especially *sp*^*2*^-bonded carbon allotropes like graphene [25,26] and carbon nanotubes [27]. It provides information on structure, disorder, doping and functional groups of the carbon nanotubes. The Raman spectra of the CNTs before and after acid-treatment showed the typical *G*, *D*, and *G'* band of graphitic materials (see S2 Fig). The *G* band is the primary Raman-active mode in graphite and represents the vibrational behavior of *sp*^*2*^-bonded carbon atoms in quasi-planar sheet configuration within the carbon nanotubes. It occurs at about 1582 cm^−1^. The *D* band appears at 1350 cm^−1^ and is known as disorder or defect mode. This mode is ascribed to the open ends of the carbon nanotubes and to defects within the tube. The *G'* mode (~2700 cm^−1^) is the overtone of the *D* band which is why it is commonly also named the *2D* band. The ratio of the intensities of the *G* and *D* bands is often used to evaluate the quality in disordered carbon materials [25,26,28]. Multi-wall nanotubes typically show a high intensity of the *D* band (see S2 Fig) compared to single-wall nanotubes, because of the large number of concentrically wrapped graphitic layers which results in a high defect density. In the spectra of single-wall nanotubes (see S2 Fig) another series of bands appears at low wavenumbers, ascribed to radial breathing modes and designated as RBM bands. These bands are unique for single-wall nanotubes and correspond to the radial contraction and expansion of the tubes. The wavenumbers of these bands can provide information about the diameter of the tubes and their aggregation state.

From the intensity ratio of the respective *D* and *G* bands, the in-plane coherence length *L*_G_, which provides information of the density of defects within the carbon nanotubes, can be calculated according to ref. [29]. The value is indicative for the degree of graphitization of the CNTs [30] and can give hints for the presence of functional groups [31]. The values of *L*_G_ (Table 3) prior to and after the purification show that the acid treatment of the carbon nanotubes lead to decreased values of *L*_G_. This is a sign for an increased number of defects and can be interpreted as a successful modification with carboxylic groups due to the treatment with nitric acid [31]. This concurs with the good dispersibility of the modified CNTs in water after the purification. By contrast, non-modified and as-received carbon nanotubes respectively, are completely non-dispersible in water [17].

**Table 3:** Calculated crystallite sizes *L*_*G*_ for as-received and purified CNT powders. The listed values are the mean of three independent measurements and the corresponding standard deviation.

tradename	CNT species	*L*_*G*_ / nm	
		as-received	purified
Baytubes C70P	MWNT	17 ± 2	20 ± 2
Fraunhofer SWNT	SWNT	518 ± 12	277 ± 8
SWeNT CG200	SWNT	170 ± 24	110 ± 21

### Carbon nanotube films

Carbon nanotube films on platinum substrates were manufactured from aqueous dispersions of the different CNTs using an automated spray-coating process. Photographic and microscopic images show a homogeneous coating of the entire substrate surface (S3 Fig). The edges of the substrates were not coated and expose bare platinum due to the geometry of the sample holder employed.

**Fig 2:**
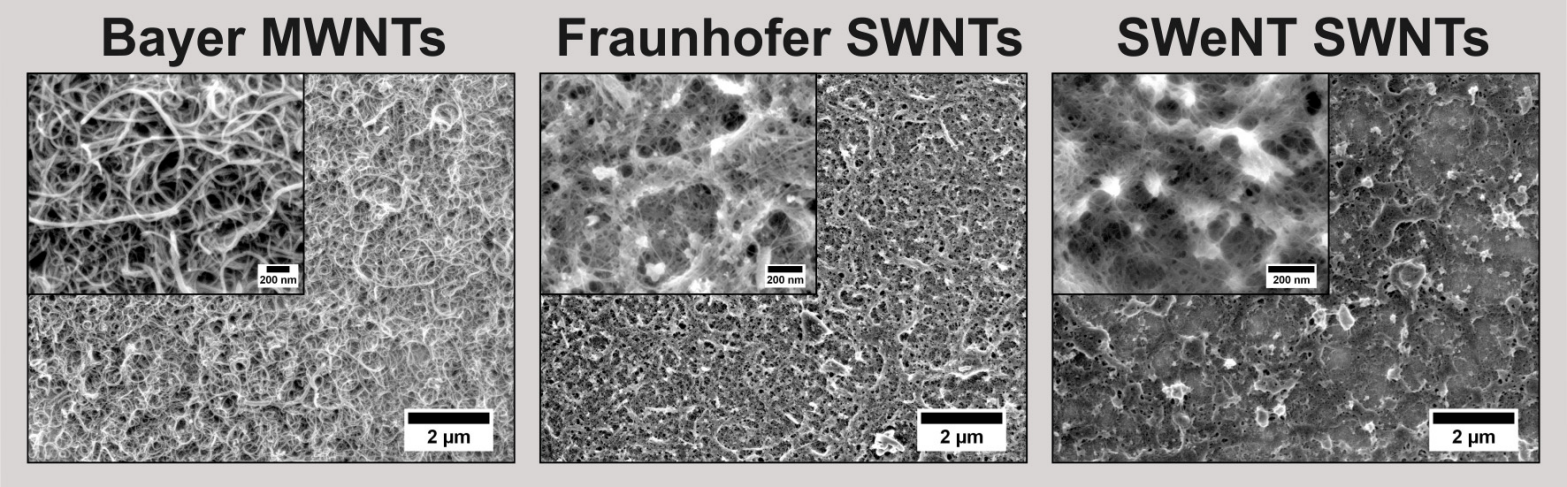
**SEM images of carbon nanotube films on platinum substrates: Bayer MWNTs, Fraunhofer SWNTs, SWeNT SWNTs (*left to right*).** The low magnifications show homogenously coated samples; in the higher magnifications in the insets, the randomly aligned networks of the carbon nanotubes become visible.

Fig 2 shows SEM images of the coatings. At low magnifications, homogenously coated areas are apparent on the micrometer scale, and no impurities are visible for Bayer MWNTs and Fraunhofer SWNTs. At higher magnifications, the impurities in the SWeNT SWNT-derived film become even clearer visible, but also the Fraunhofer-SWNT-based film shows the presence of small inorganic particles, probably remaining catalyst particles. In contrast to the CNT powder (see Fig 1), SEM images of the Fraunhofer SWNT-derived film exhibit no nanotubes with large diameters, probably due to the poor dispersibility of such large nanotubes in water. In all cases, the carbon nanotubes do not show a preferred orientation, but form a randomly aligned network of interwoven nanotubes (Fig 2).

The SEM images of the films indicate a rough surface on the nanometer scale, which is verified by confocal microscopy (S4 Fig). To determine the film thickness, the CNT coatings were partially removed prior to confocal microscopic investigation. The well-defined edges allow the determination of the thicknesses of the CNT coatings. Because of the rough surface of the coatings, only a general range of values of of 60 to 130 nm can be given. The arithmetic average of the 3D roughness *Sa* shows values of 30 up to 40 nm, so in relation to the absolute film thicknesses the surfaces are very rough.

X-ray photoelectron spectroscopic (XPS) investigations on the CNT films provide information on the elemental composition of the surface of the coatings. The surface compositions of the different CNT films determined via XPS are listed in Table 4. High oxygen contents of the CNT coatings provide further evidence for the successful modification with oxygen-containing functional groups [32]. The nitrogen content is relatively low although nitric acid was used for the purification process. No transition metals, as they were found in the ICP-OES analysis of the CNT dispersions, could be detected on the surface of the coatings. Either the metal contents are too low for this analysis technique, or the particles containing the metals are not at the surface of the coatings due to encapsulation in the CNTs.

**Table 4:** Elemental compositions of the surfaces of the prepared CNT films determined via X-ray photoelectron spectroscopy (XPS). Only elements with a percentage larger than 0.5 at% are listed.

CNT film	surface composition / at%			
	C	O	N	Ca
Bayer MWNTs	80.0 ± 8.2	17.1 ± 6.9	1.5 ± 0.9	0.7 ± 0.5
Fraunhofer SWNTs	73.7 ± 1.2	22.9 ± 1.0	2.2 ± 0.2	0.6 ± 0.1
SWeNT SWNTs	79.2 ± 7.5	17.7 ± 6.7	1.7 ± 0.6	0.7 ± 0.5

Carbon nanotube coatings of electrodes [10,18,33] or even pure carbon nanotube electrodes [34] are in focus for neural interface application. Keefer *et al*. showed in 2008 that carbon nanotube coating of multi-electrode arrays enhances the recording quality and electrical stimulation *in vitro* in neuronal cell cultures and *in vivo* in rats and monkeys by decreasing the impedance and increasing charge transfer [10]. Baranauskas *et al*. could determine an improvement of the signal-to-noise ratio for the recording of neuronal signals due to the reduced impedances by a CNT coating on neural microelectrodes [18]. Moreover, reduced impedances lead to a more focused stimulation of the target cells.

**Fig 3:**
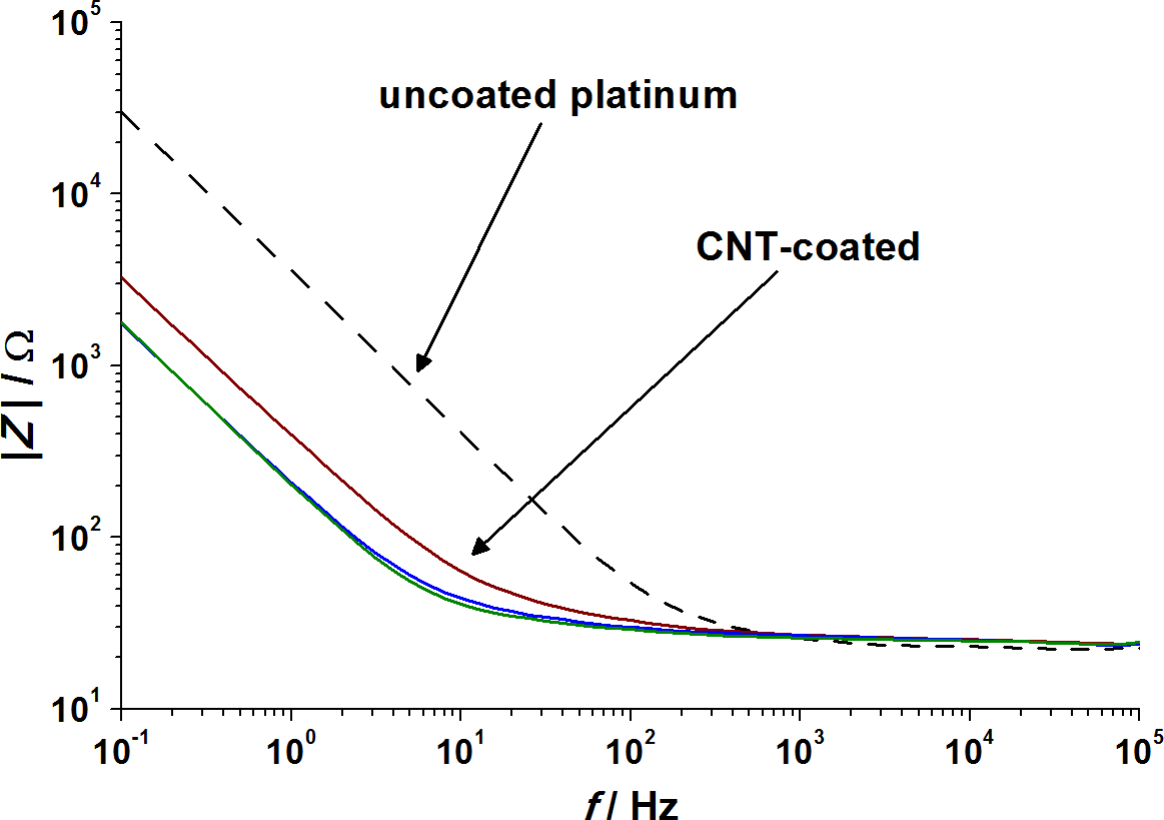
Impedance spectroscopy scan of the CNT-coated platinum substrates in comparison to a non-coated platinum electrode. *Red*: Bayer MWNTs, *blue*: Fraunhofer SWNTs, *green*: SWeNT SWNTs, *dashed black*: uncoated platinum substrate.

In view of potential applications of CNT-coated neuronal electrodes, the electrical properties of the CNT films on platinum surfaces were investigated via impedance spectroscopy. The scans (Fig 3) show for the non-coated electrode as well as for all CNT-coated electrodes a typical increase of the impedance at low frequencies, whereas the impedance is almost constant for the high frequency range due to the uncompensated resistance of the electrode, which is the same for CNT-coated and non-coated electrodes [15]. The CNT coatings entail a drop of the impedance in the low frequency range (below 10^2^ Hz) in comparison to the non-coated bare platinum electrode. This impedance decrease, corresponding to about one order of magnitude, is found for all three different types of CNT coatings. The drop of the impedance is mainly caused by the increased surface of the electrode by the coating with CNTs. Similar impedance decreases have been reported for other CNT coatings and films [10,18,33]. Compared to common microelectrodes [10,33] the absolute impedance values shown here are low, caused by the comparatively large area of the electrode contacts (1.5 cm^2^).

Resulting capacitance values (for the calculation model see S5 Fig) are listed in Table 5 [35,36]. The CNT coatings lead to more than tenfold higher capacitances of the electrodes (300–600 μF∙cm^−2^) compared to uncoated platinum electrodes (20–30 μF∙cm^−2^), with the films containing single-wall nanotubes having twice higher values than those prepared from multi-wall nanotubes. Although the decrease of the impedance due to the CNT coating and the resultant higher capacitances of the CNT-coated electrodes were clearly evident, even larger reductions of impedances were reported from other research groups for electrode modification methods with CNTs [10,37]. However, these results refer to small electrodes where the initial impedances of the uncoated electrodes are very high. When large-scale electrodes are used for modification, for example by placing gold nanopillars on the surface, similar results to ours were obtained [35,38].

**Table 5:** Calculated values for resistances and capacitances of the prepared CNT films. The evaluation procedure is described in the Supporting Information. α is an exponent to compensate for non-homogeneity in the system, as caused, e.g. by a double-layer capacitance of a porous or rough surface. The calculation was based on a typical measurement of one sample for each type of coating or uncoated platinum, respectively.

	Bayer MWNTs	Fraunhofer SWNTs	SWeNT SWNTs	uncoated platinum
resistance / Ω	27.1 ± 0.3	27.0 ± 0.3	26.5 ± 0.2	24.2 ± 0.5
normalized capacitance / μF∙cm^‒2^	335 ± 6	617 ± 11	610 ± 10	27.4 ± 0.5
exponent α / -	0.88 ± 0.01	0.89 ± 0.01	0.92 ± 0.01	-

Basic observations regarding the mechanical stability and the stability against aqueous solutions can be made by the optical investigation of the carbon nanotube films after impedance spectroscopic measurements and after the cell culture investigations. In both cases, no changes were visible after the experiment, which is also true when the carbon nanotube films on platinum were exposed to ultrapure water for one week. Via turbidimetry measurements using an UV-vis spectrometer, no increased turbidity of the water was detectable, although this method is able to substantiate a CNT mass fraction of only 10^−5^% in a CNT dispersion. Additionally, the comparison of SEM images of the samples prior to and after the cell culture experiments with NIH3T3 fibroblasts showed no significant changes in the morphology of the CNT coatings (see S6 Fig and S7 Fig). Thus, a general stability of the carbon nanotube films against water and cell culture medium can be stated. Even the detachment of the fibroblasts with trypsin/EDTA solution had no influence on the coatings. With regard to the mechanical stability, the carbon nanotube films easily withstand the regular handling (except for directly touching or rubbing on the CNT-coated sample surface) during the coating process and the subsequent measurements without scratches, spallings or other optical damage. Obviously, this might not be comparable with the requirements that the coatings would have to comply for a potential application in the cochlear implant. During implantation the coatings might have to withstand strong shear forces, although for some types of implants the contacts are somewhat recessed into the silicone casing. In any case, further investigations on the mechanical stability of the CNT coatings would be crucial to evaluate the suitability of the coatings for this potential application.

### Cell culture investigations

The cytocompatibility and biocompatibility of carbon nanotubes and coatings of carbon nanotubes are still under discussion [39]. Due to different fabrication processes (types and amounts of employed catalysts) and varying properties of carbon nanotubes (lengths, diameters, modifications, purity, degree of graphitization), a general statement regarding the biocompatibility of CNTs cannot be provided at present, which is why further studies are of prime importance. Here, we present tests on the general cytocompatibility of our preparations of CNTs using NIH3T3 fibroblasts as a standard cell line of robust cells. More importantly, we also present the first study of the interaction of CNT films with spiral ganglion cells, including the specific glutamatergic neurons of the inner ear. This is important in view of a possible application of our CNT films on cochlear electrodes. It is to be noted that our CNT preparations and correspondingly also the film are free from dispersing agents like polymers.

#### Fibroblast cell culture studies

**Fig 4:**
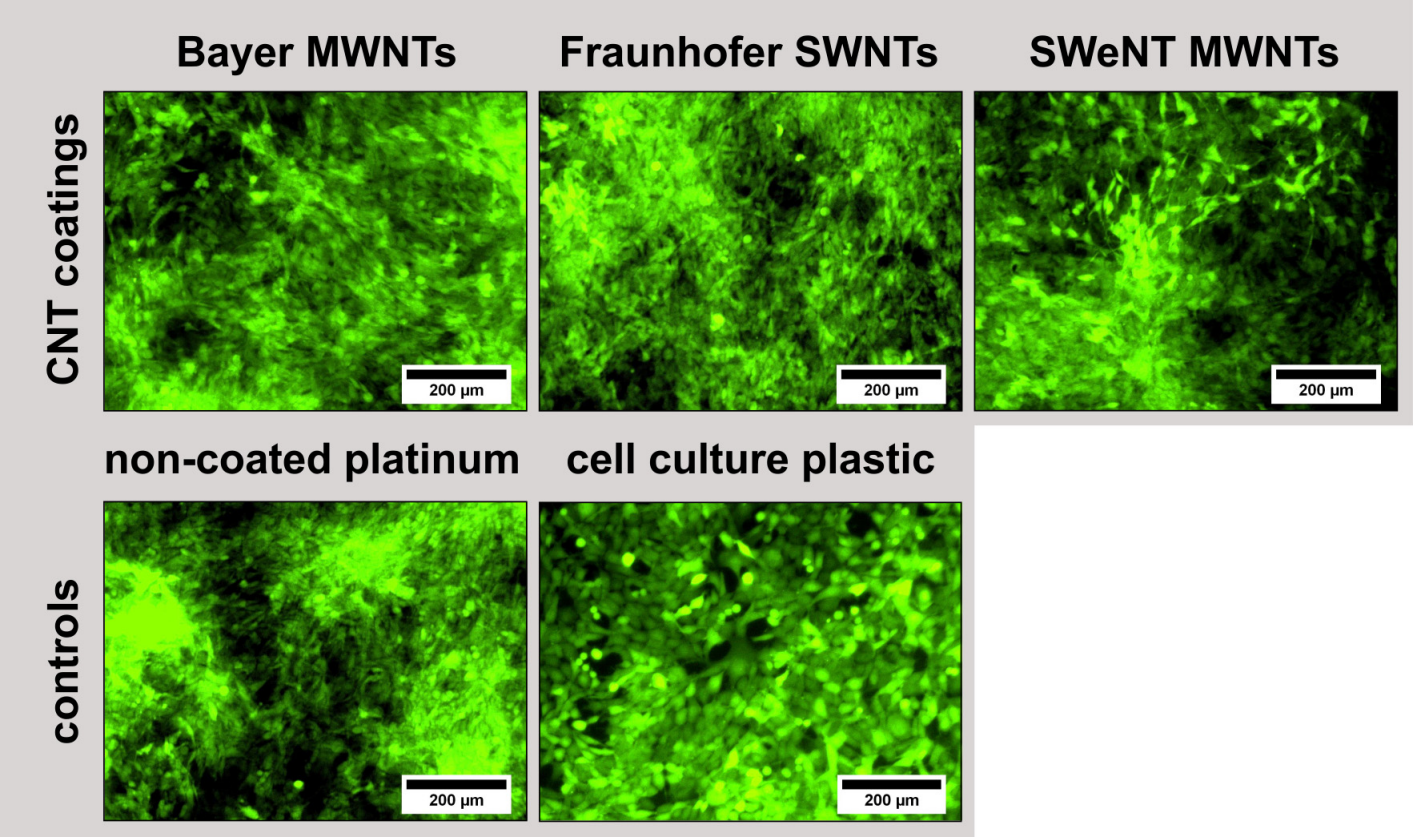
Fluorescence microscopic images of GFP-expressing NIH3T3 fibroblasts (*N* = 4, *n* = 3) after four days of cultivation on CNT-coated platinum substrates as well as on non-coated platinum and on cell culture plastic.

**Fig 5:**
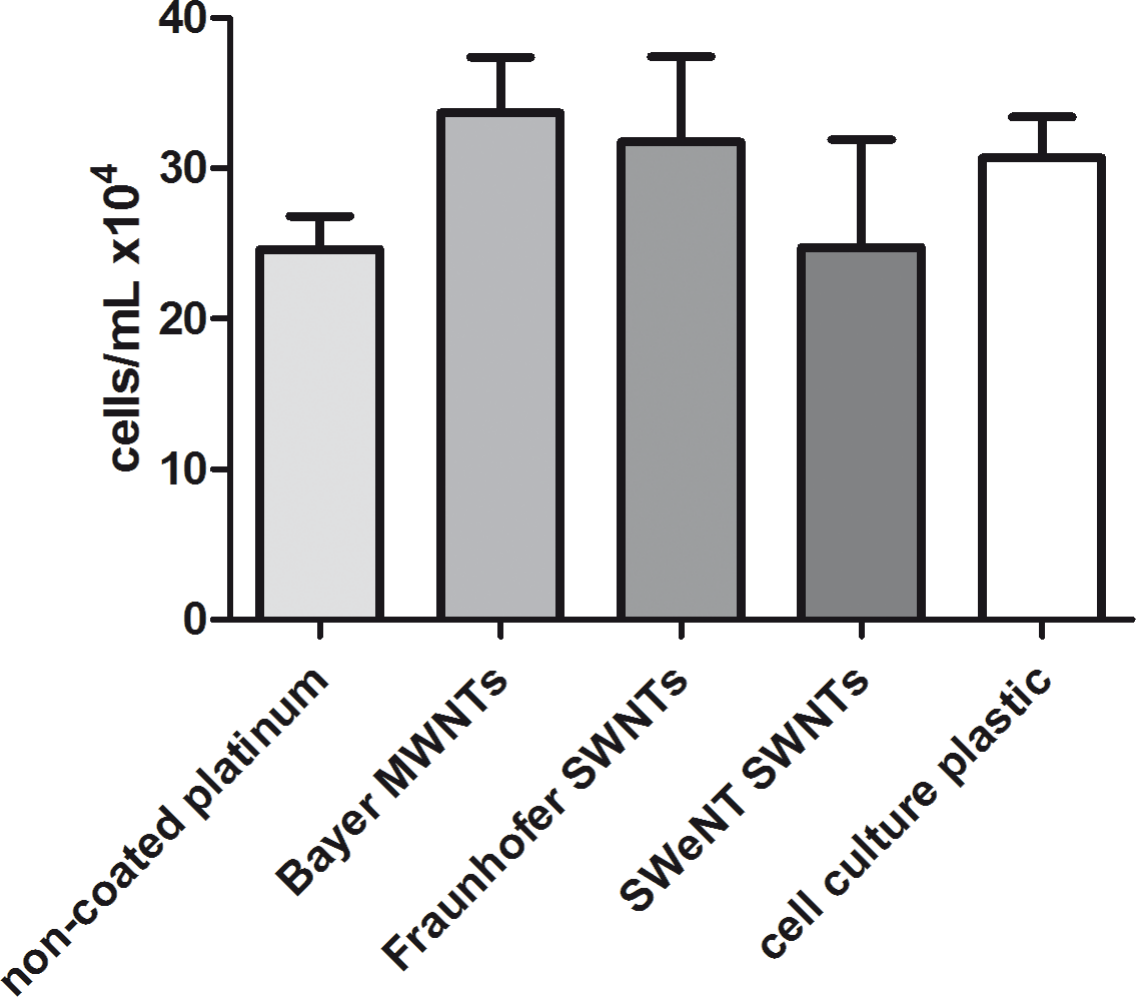
Comparison of the determined cell numbers of NIH3T3 fibroblasts (*N* = 4, *n* = 3) grown on CNT-coated and non-coated platinum substrates, respectively, plus on cell culture plastic after 4 days of cultivation. Values are given as mean ± standard error of the mean; *N* = 4, *n* = 3; One-way ANOVA with Bonferroni's multiple comparison test was used to compare means, which were not significant.

Fibroblasts were cultivated on the CNT films to obtain general information with regard to the cytocompatibility of the CNT coatings on platinum substrates. The cell viability and proliferation of GFP-expressing NIH3T3 fibroblasts were investigated. Fluorescence microscopic investigations after four days of cultivation showed good viability and proliferation for all CNT-coated samples, comparable to the non-coated platinum and cell culture plastic (Fig 4). Determined cell numbers of the Bayer MWNT and Fraunhofer SWNT films as well as of the cell culture plastic were all at the same level and indicated an excellent cytocompatibility of these CNT coatings for fibroblasts (Fig 5). The SWeNT SWNT films showed cell numbers on the same level with the non-coated platinum, still indicating a good cytocompatibility as well. However, these CNT films do not support the cells as well as the cell culture plastic and the other coatings. Possibly, the cells did not adhere as well as on the other two types of CNT films and detached easier. In general, all of the different coatings showed a favorable cytocompatible behavior, which is comparable to uncoated platinum surfaces and to the. cell culture plastic.

#### Spiral ganglion cell cultures

An important goal is to advance CNTs for neural interface applications in the auditory system. One potential strategy could be the coating of platinum surfaces of the electrodes of neuroprosthesis with CNTs. Thus, the specific interaction of the primary auditory neurons of the inner ear, i.e. the spiral ganglion neurons (SGNs), with the CNT coatings is of great importance. For this purpose, the cytocompatibility of the CNT coatings was tested on spiral ganglion cell cultures.

**Fig 6:**
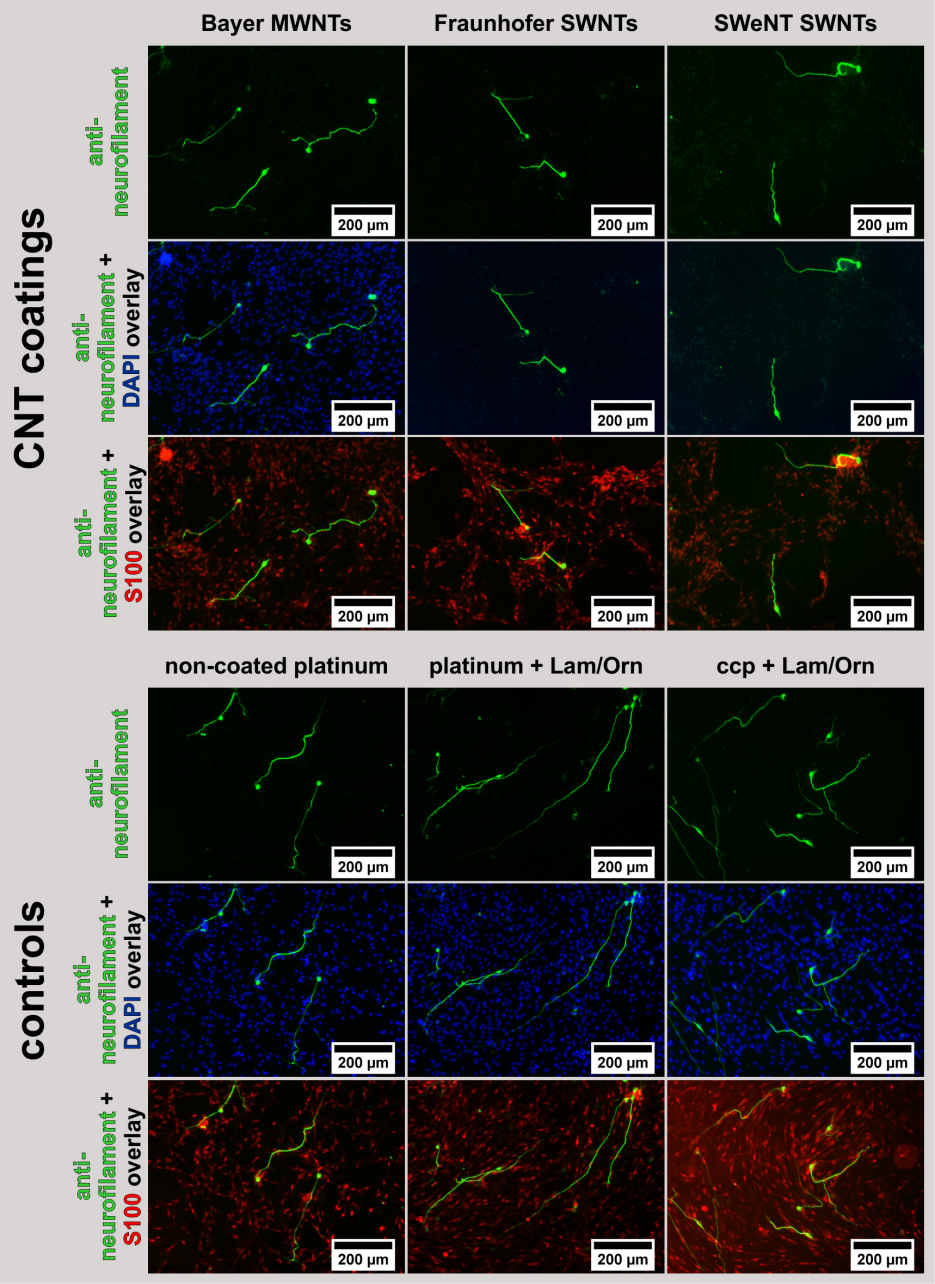
Immunofluorescence staining of spiral ganglion cell cultures cultivated for 48 h on CNT-coated platinum samples and laminin- and poly-D/L-ornithine-coated control surfaces (*N* = 4, *n* = 2). The spiral ganglion neurons (*green*) of the mixed SGC culture were neurofilament positive. Additionally, cell nuclei (*blue*) were fluorescence-stained with 4',6-diamidin-2-phenylindol (DAPI) and the glial cells (*red*) were stained with S100. These images are depicted as overlay combined with the anti-neurofilament images; ccp: cell culture plastic.

Representative microscopic images of the SGCs stained immunocytochemically for neurofilament after two days of cultivation showed surviving neurons with neuronal outgrowth for all samples (Fig 6). Laminin- and poly-D/L-ornithine-coated (Lam/Orn) platinum and cell culture plastic, showed the highest number of neurons with the longest neuronal outgrowths. These two conditions were an indicator that the SGC isolation was good and the cell culture grew as usual. On bare platinum, shorter and less branched neurites were observed when compared to the laminin- and poly-D/L-ornithine-coated samples. This indicates a less favorable interaction between the SGN and pure platinum. Neurite outgrowth from SGNs was also observed on CNT-coated platinum samples. The neurons were comparable in size and number with the neurons on the non-coated platinum surface and showed a healthy morphology. However, fewer neurons attached and survived on the SWNT-based coatings.

**Fig 7:**
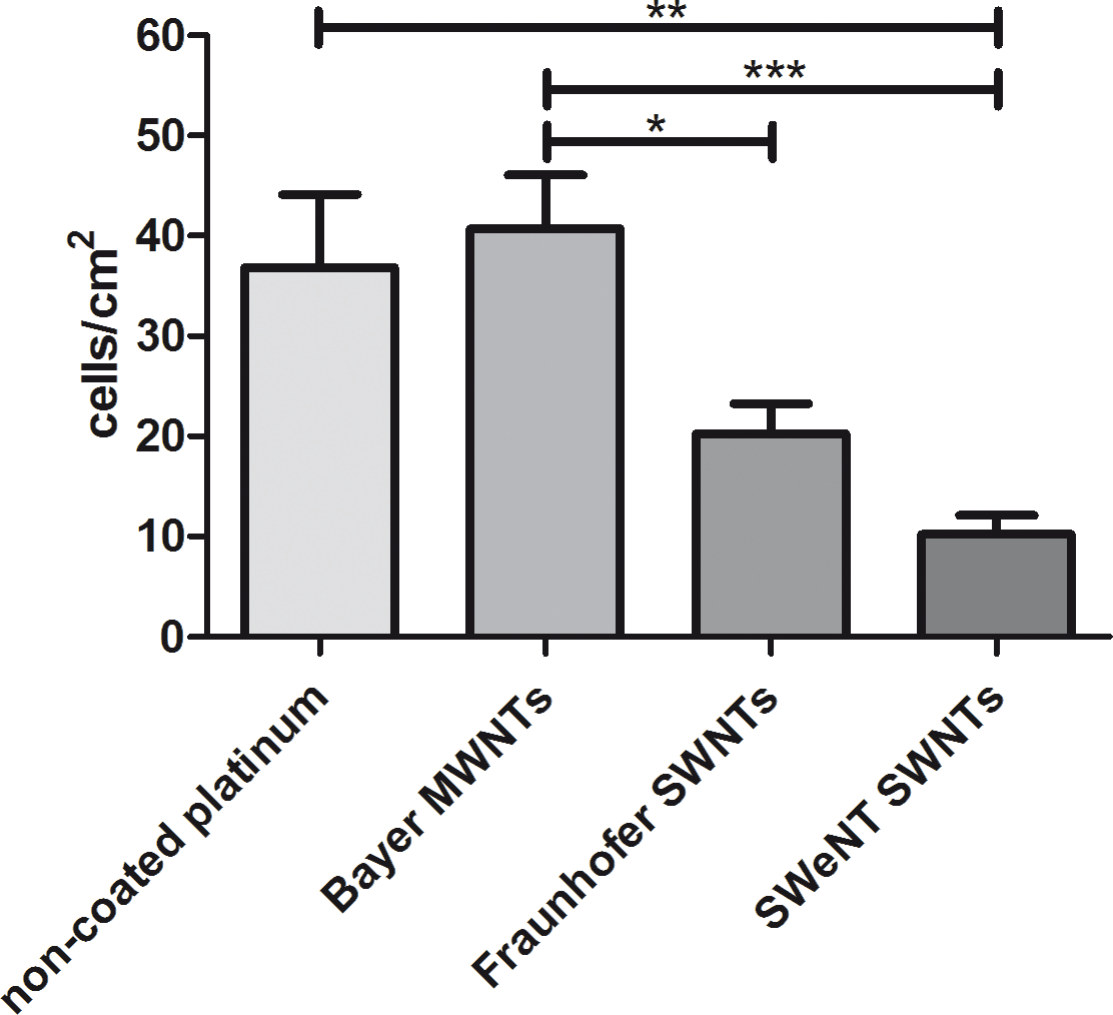
Comparison of the number of spiral ganglion cells (*N* = 4, *n* = 2) after two days of cultivation. Cells were grown on CNT-coated platinum substrates as well as, on non-coated platinum. Neurofilament-positive cells were counted under the fluorescence microscope. Values are given as mean ± standard error of the mean; *N* = 4, *n* = 2. Asterisks indicate the significance of the different results. Statistical assessment was performed using one-way ANOVA with Bonferroni's multiple comparison test (*p < 0.05; **p < 0.01; ***p < 0.001).

The numbers of survived SGNs were determined under the fluorescence microscope. The values for cells per cm^2^ are shown in Fig 7 and confirm the results gathered from the fluorescence microscopic investigations. The numbers of survived SGNs for the laminin- and poly-D/L-ornithine-coated platinum (103 cells·cm^−2^) and cell culture plastic (88 cells·cm^−2^) were the highest (see S8 Fig). This result was expected, because the poly-D/L-ornithine and laminin coating provides ideal growing conditions for neurons (extracellular proteins allow neurons to adhere) and served here as a quality control for the SGN cell culture. The cell densities depicted in Fig 7 for the investigated CNT-coated samples and uncoated platinum are less than half as those of the laminin- and ornithine-coated samples. More relevant is the comparison of the investigated samples among each other. CNT films consisting of multi-wall nanotubes (Bayer MWNTs, 40 cells·cm^−2^) showed a comparable number of SGNs as the non-coated platinum samples (36 cells·cm^−2^). Coatings of single-wall nanotubes resulted in consistently lower survival of the neurons, although a direct cytotoxic effect for the SWNT-based coatings was not demonstrated in the screening experiments with the fibroblast cell line. The number of counted SGNs was significantly lower for Fraunhofer SWNTs (20 cells·cm^−2^) and highly significantly reduced for the SWeNT SWNTs (10 cells·cm^−2^) when compared to the Bayer MWNTs (40 cells·cm^−2^). The number of SGNs on SWeNT SWNT coatings was also significantly reduced in comparison to the non-coated platinum. Among the tested coatings, the coating with Bayer MWNTs allowed similar attachment and growth of SGNs as bare platinum and was therefore the best tested CNT coating.

In order to survive in cell culture, SGNs must be offered an ideal environment for cell adhesion as well as a sufficient amount of bystander cells. In the human cochlea, it has been shown that the unmyelinated cell somata of the SGNs are surrounded by satellite ganglion cells [40]. After degeneration of the peripheral process, the dendrites of the SGNs are consolidated through a concerted action of satellite glial cells involving Cx43-mediated GJ signaling, thus preventing SGNs from degeneration [41].

The isolation of SGCs leads to a mixed cell culture containing SGNs, glial cells and fibroblasts. By staining the cells with the neuron-specific anti-neurofilament antibody, the SGNs can be distinguished from the bystander cells. By additionally staining with 4',6-Diamidin-2-phenylindol (DAPI), the cell nuclei of all cell types should be detectable. In the merged images of DAPI and neurofilament staining (Fig 6), the DAPI staining of cells on the SWNT coatings is hampered: No blue-stained nuclei are apparent. However, additional staining with an antibody specific for glial cells (S100) shows the presence of these additional bystander cells. SWNTs are known to influence biological tests as for example the MTT cytotoxicity assay [42,43], by interacting with the reactants or products of staining reactions. A similar case appears to prevail here. Therefore, it is of great importance to apply different assays for determining the outcome of biological tests carried out in the presence of highly surface-active materials like the purified and funtionalized CNTs used here.

Xie *et al*. (2006) have proposed strong interactions at the nanoscale between dorsal root ganglion neurons and functionalized MWNTs. The functional groups of the CNTs are supposed to function as anchor points and enhance the adhesion of the neurons as well as to promote neurite outgrowth [15]. Although the preparation procedure applied to the CNTs by Xie *et al*. is similar to the one used here, we were not able to make similar observations for spiral ganglion neurons. Moreover, they showed that on unfunctionalized CNT surfaces the neurites were much shorter than on functionalized CNTs. Kim *et al*. reported in 2014 that neurons cultivated on CNT-coated surfaces initiate their neurite outgrowth one to two days later than on standard coatings [16].

#### Carbon nanotube supernatants

**Fig 8:**
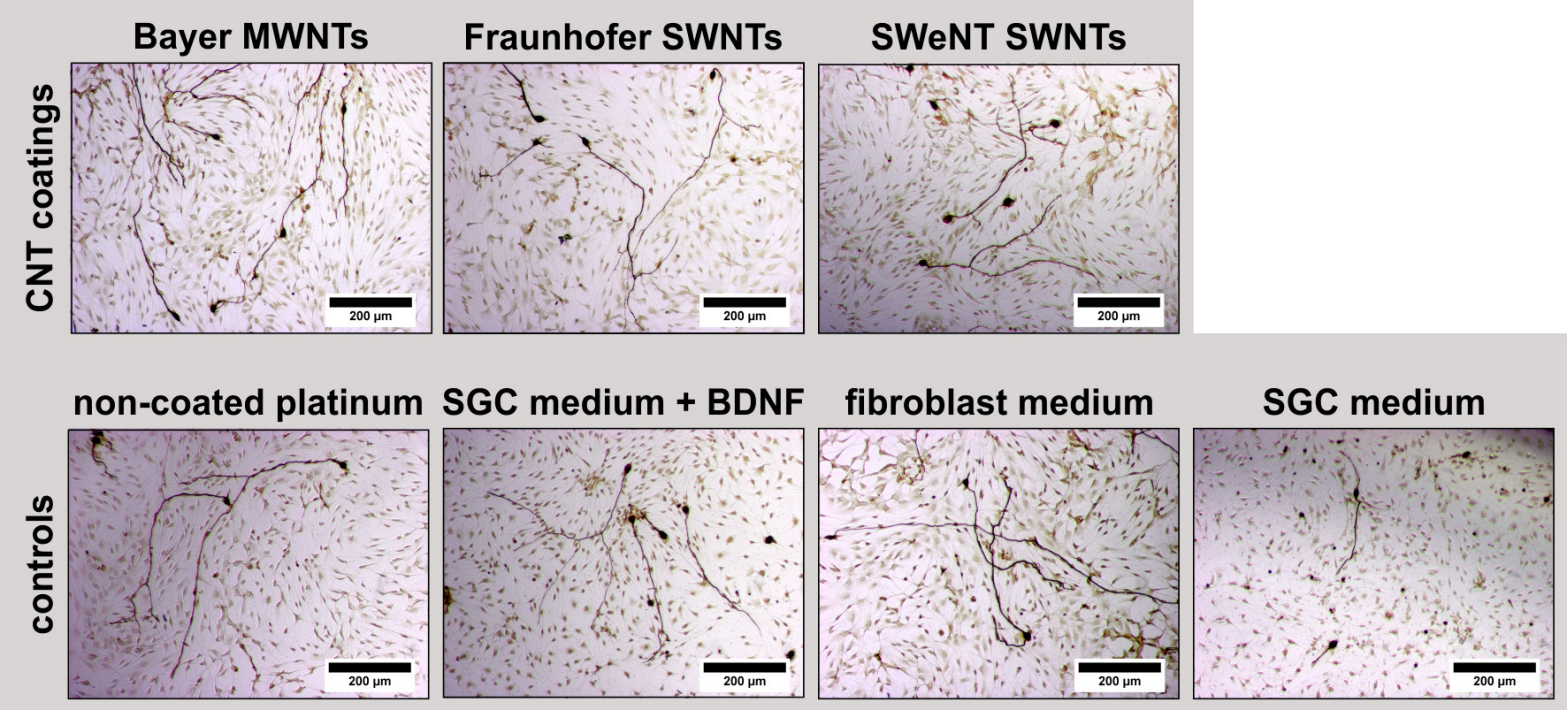
Transmission light microscopic images of spiral ganglion cells (SGCs) (*N* = 1, *n* = 9) after two days of cultivation. The samples differed in the media composition. The media contained supernatants from incubating CNT-coated (Bayer MWNT-coating, Fraunhofer SWNT-coating, SWeNT SWNT-coating) and non-coated platinum substrates in serum-containing medium. For comparison, SGCs were also cultivated in SGC medium supplemented with BDNF (BDNF control) and serum-containing medium (FCS control) as well as in serum-free SGC medium (medium control).

The spiral ganglion cell culture experiments on CNT films showed limited neuronal adhesion, neurite extension and survival on single-wall CNT-based coatings. The inferior results obtained with the two single-wall CNT-based coatings may be related to either remaining catalyst particles or inorganic salts from the purification procedure. Therefore, additional experiments were carried out with SGN cell cultures. In these experiments, supernatants were used which were obtained by incubation of corresponding film samples in serum-containing cell culture medium, using both CNT-coated and non-coated platinum. The supernatants collected were subsequently added to the SGC culture. The cells were cultivated for two days in a 1:1 mixture of SGN medium (serum-free) and the collected supernatants (serum-containing). Representative transmission light microscopic images show the proliferation behavior of SGNs and their neuronal outgrowth (Fig 8). Morphologically, there is no difference between all tested conditions except for the serum-deprived condition (medium control) showing only few surviving neurons.

**Fig 9:**
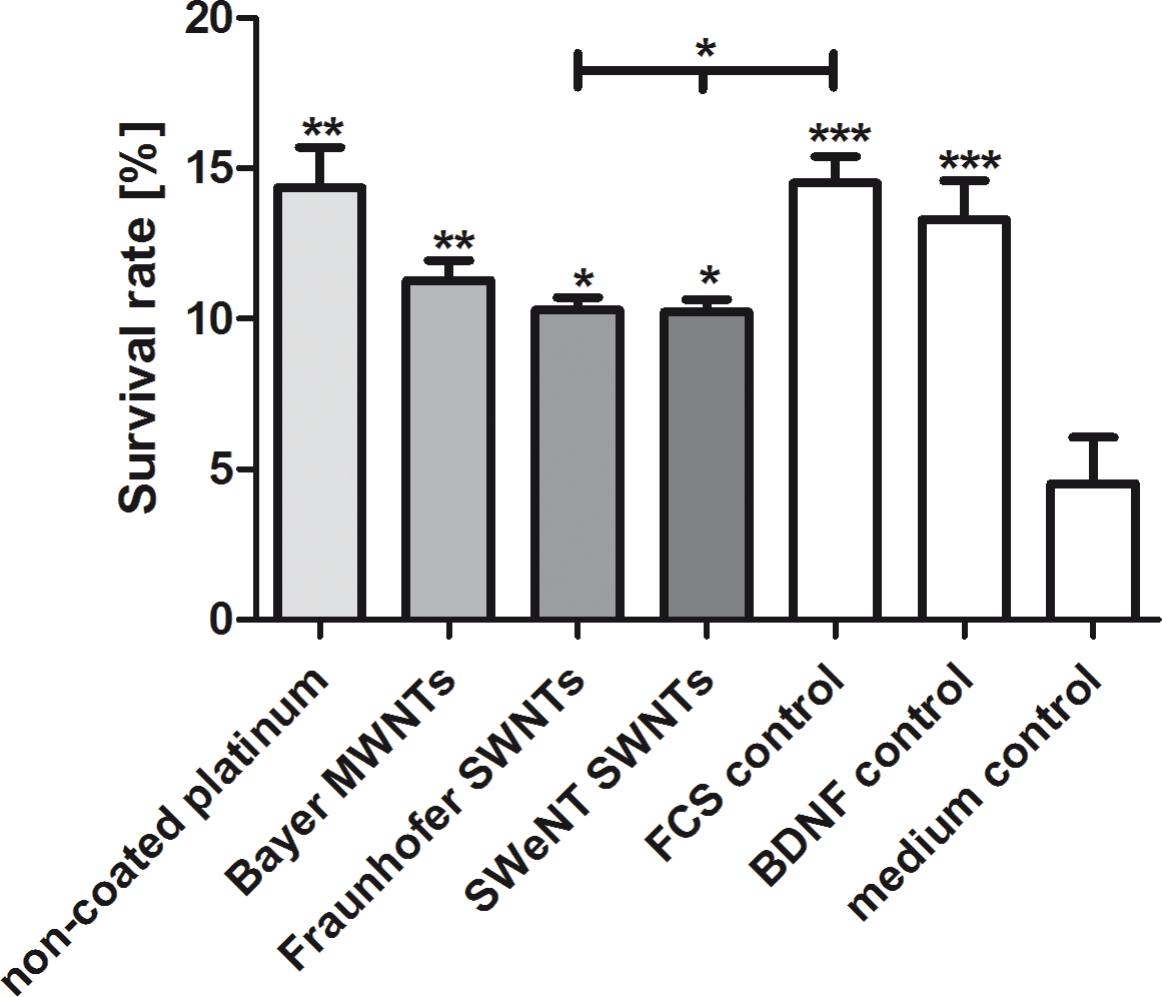
Survival rate of spiral ganglion neurons after two days of cultivation (*N* = 1, *n* = 9). The survival rates were determined by the amount of neurons in relation to the seeding control after cultivation in supernatants obtained from different CNT-coated samples. Supernatants were obtained from CNT-coated (Bayer MWNT-coating, Fraunhofer SWNT-coating, SWeNT SWNT-coating) and non-coated platinum substrates incubated in serum-containing medium (FCS control). SGC medium supplemented with BDNF (BDNF control) and serum-containing medium (FCS control) as well as SGN medium without the addition of any growth factors (medium control) were used as controls. Values are given as mean ± standard error of the mean (*N* = 1, *n* = 9). Asterisks indicate the significance of the survival rates of the different conditions compared to the negative control. Statistical assessment was performed using one-way ANOVA with Bonferroni's multiple comparison test (n.s. = not significant, *p < 0.05; **p < 0.01; ***p < 0.001).

After quantification, a significantly increased survival rate of SGNs cultivated in supernatants collected from the different coatings was observed in all tested conditions when compared to the medium control. Supernatants of single-wall CNT-coated (Fraunhofer SWNT and SWeNT SWNTs) samples resulted in significantly reduced survival rates when compared to the FCS control. Interestingly, the survival rates of SGNs cultivated in serum-containing supernatants obtained from uncoated platinum were comparable to the FCS control and BDNF control. Whether these results are due to soluble toxic components released by the CNT coatings or whether this is due to the capture of nutritional components of the serum by the CNT coatings cannot be distinguished in this experimental setting. As the failure of the DAPI staining method on the SWNT-based films indicates, single-wall nanotubes show strong interactions with molecules presented to them in solution. Since the neuronal survival rates obtained in the cultures testing the supernatants (Fig 9) are much better than after direct cultivation of the neurons on the coatings (Fig 7), we assume that the hindered adhesion and differentiation on the substrate—and not indirect toxic effects—are the main reason for the reduced number of cells on the CNT-coated samples depicted in Fig 7. Here, the survival rates were comparable to uncoated platinum, indicating that the neurons require an adequate substrate—as offered for example by the coating with laminin and poly-D/L-ornithine—for improved survival.

## Conclusions

In this study we have combined the chemical processing of as-delivered CNTs, the fabrication of coatings on platinum, and the characterization of the electrical properties of the coatings as well as a general cytocompatibility testing and cell culture investigations with spiral ganglion neurons. The chemical purification and surface modification of the different CNTs was successful and allowed the production of long-term stable aqueous CNT dispersions free from dispersing agents. This offered the possibility to coat platinum substrates via the cost-effective process of automated spray-coating which is also suitable for the preparation of large numbers of samples. Uniform, homogeneous coatings of modified CNTs on platinum have been obtained with very good reproducibility. Such coatings show enhanced electrical properties as they would be preferred for applications in neuronal electrodes. A generally favorable cytocompatibility of the CNT coatings was established using cell culture investigations with NIH3T3 fibroblasts. For spiral ganglion neurons the CNT coating with the Bayer MWNTs on platinum showed similar numbers of SGNs as the uncoated platinum. By contrast, the two CNT coatings with SWNTs showed significantly reduced numbers of SGNs when compared to the MWNT coatings and the non-coated platinum. Noteworthy, an especially favorable effect on the biocompatibility for neuronal cells–which was observed for other types of CNTs and with other types of neurons [9,13,14]–could not be established for SGNs. In this context, it has to be considered that SGNs are extremely sensitive cells. For an increased survival they would possibly need additional incentives, like a coating with laminin or the presence of neuroprotective factors. However, in view of the general uncertainty regarding medical applications of CNTs [39], especially if it cannot totally be excluded that these become mobile, it appears doubtful whether applications of CNT coatings on neuronal electrodes will be successful. Our investigations further showed that care must be taken when well-established biological assays like the DAPI staining are applied on materials with large and highly active surfaces like single-wall carbon nanotubes. Due to the large specific surface area and the reactivity of the surface, chemicals may become bound to the material and can thus be effectively removed from the test system [42,43].

## Supporting Information

S1 Fig Thermogravimetric measurements of the CNT material prior to and after the acid treatment. The as-received CNT materials are depicted in dotted lines and the purified in solid lines. The measurements were used to determine the residue after the thermal treatment in flowing air.
(TIF)

S2 Fig Raman spectra for as-received and purified CNT materials. The as-received CNT materials are depicted in dotted lines and the purified in solid lines. The spectra were used to identify the typical G, D, G' bands of graphitic materials and their intensities to calculate the *L*_*G*_ values.
(TIF)

S3 Fig **Photographic images of an uncoated platinum substrate (*far left*) and (*further from left to right*) carbon nanotube films on platinum substrates: Bayer MWNTs, Fraunhofer SWNTs, SWeNT SWNTs.** The edges of the substrates were not coated and expose bare platinum due to the geometry of the sample holder employed.
(TIF)

S4 Fig **Three-dimensional image of the confocal microscopic investigation of the CNT-coated platinum substrates: Bayer MWNTs, Fraunhofer SWNTs, SWeNT SWNTs (*left to right*).** Parts of the CNT coatings were removed mechanically. The formed edges between coating and substrate were used to determine the film thickness of the coatings via confocal microscopy scans. Dark blue areas in the three-dimensional images represent the bare plane platinum surface of the substrate. Green, yellow and orange colors respectively denote different heights of the coating.
(TIF)

S5 Fig **Equivalent circuits: R-C serial circuit for uncoated platinum electrodes (*left*), R-CPE serial circuit model for CNT-coated electrodes (*right*).** To calculate the capacitance values of the electrodes, equivalent circuits consisting of a constant phase element *CPE* and or a capacitor *C* together with a resistor *R* were chosen. The *CPE* was used to fit the data for CNT films due to their rough and porous surface, whereas a normal capacitor was chosen to fit the data for planar platinum surfaces [35,36]. In the term for the CPE, the exponent α appears which is given in Table 4.
(TIF)

S6 Fig **Comparison of SEM images of carbon nanotube films on platinum substrates prior and after cell culture experiments with NIH3T3 fibroblastes: Bayer MWNTs, Fraunhofer SWNTs, SWeNT SWNTs (*left to right*).** The fibroblasts were detached via trypsin/EDTA solution after the cell culture experiments and prior to the SEM investigations. No changes of the CNT coatings are visible.
(TIF)

S7 Fig **Comparison of cross-section-SEM images of carbon nanotube films on platinum substrates prior and after cell culture experiments with NIH3T3 fibroblastes: Bayer MWNTs, Fraunhofer SWNTs, SWeNT SWNTs (*left to right*).** The fibroblasts were detached via trypsin/EDTA solution after the cell culture experiments and prior to the SEM investigations. No changes in film thickness of the CNT coatings are visible.
(TIF)

S8 Fig Comparison of the number of spiral ganglion cells (*N* = 4, *n* = 2) after two days of cultivation. Cells were grown on CNT-coated platinum substrates and non-coated platinum as well as, for comparison, on non-coated platinum and cell culture plastic (ccp), both coated with laminin and poly-D/L-ornithine. Neurofilament-positive cells were counted under the fluorescence microscope. Values are given as mean ± standard error of the mean; *N* **=** 4, *n* **=** 2. Asterisks indicate the significance of cell per cm^2^ of the different samples compared to the laminin- and poly-D/L-ornithine-coated platinum. Statistical assessment was performed using one-way ANOVA with Bonferroni's multiple comparison test (n.s. **=** not significant; *p < 0.05; **p < 0.01; ***p < 0.001).
(TIF)
