# Supplementary figures and images for: Coatings of Different Carbon Nanotubes on Platinum Electrodes for Neuronal Devices: Preparation, Cytocompatibility and Interaction with Spiral Ganglion Cells
Source: PLoS One. 2016 Jul 6;11(7):e0158571. doi: 10.1371/journal.pone.0158571 (PMC4934701; doi:10.1371/journal.pone.0158571)

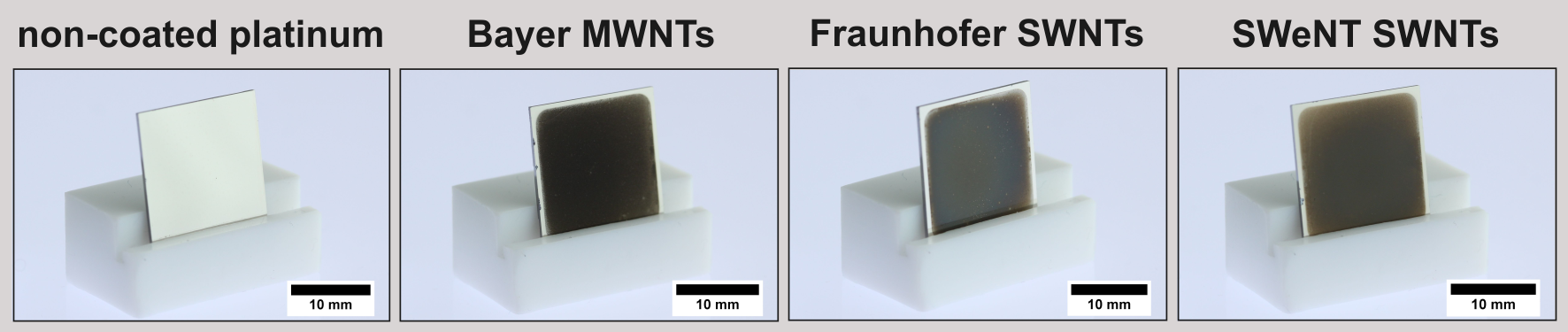

Supplement: S3 Fig — Photographic images of an uncoated platinum substrate (far left) and (further from left to right) carbon nanotube films on platinum substrates: Bayer MWNTs, Fraunhofer SWNTs, SWeNT SWNTs. The edges of the substrates were not coated and expose bare platinum due to the geometry of the sample holder employed. (TIF) [file pone.0158571.s003.tif]

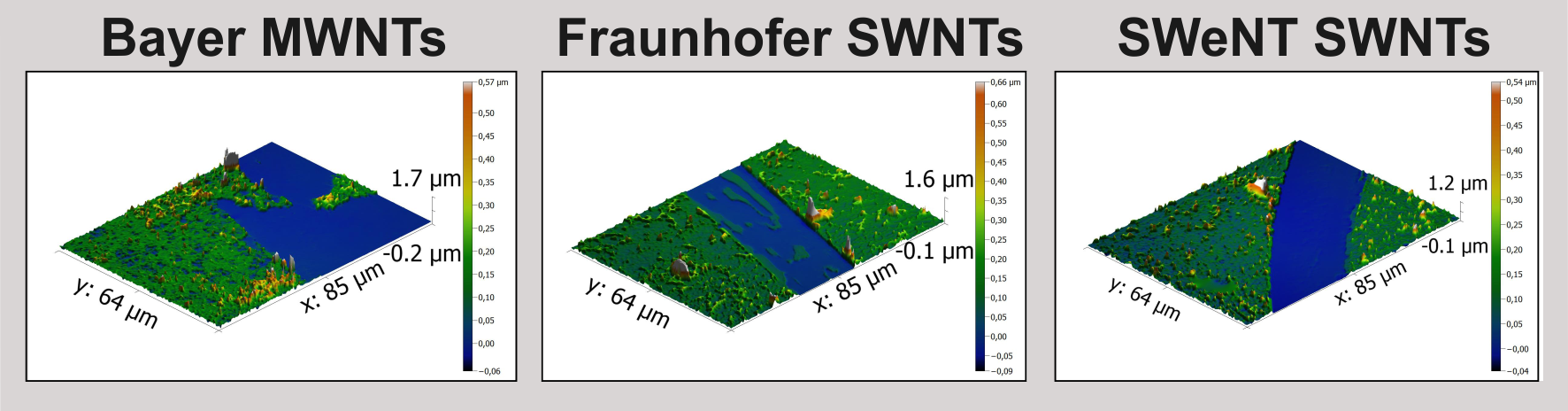

Supplement: S4 Fig — Three-dimensional image of the confocal microscopic investigation of the CNT-coated platinum substrates: Bayer MWNTs, Fraunhofer SWNTs, SWeNT SWNTs (left to right). Parts of the CNT coatings were removed mechanically. The formed edges between coating and substrate were used to determine the film thickness of the coatings via confocal microscopy scans. Dark blue areas in the three-dimensional images represent the bare plane platinum surface of the substrate. Green, yellow and orange colors respectively denote different heights of the coating. (TIF) [file pone.0158571.s004.tif]

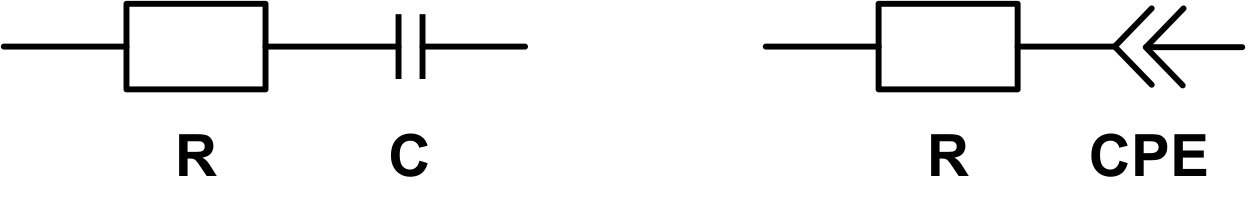

Supplement: S5 Fig — Equivalent circuits: R-C serial circuit for uncoated platinum electrodes (left), R-CPE serial circuit model for CNT-coated electrodes (right). To calculate the capacitance values of the electrodes, equivalent circuits consisting of a constant phase element CPE and or a capacitor C together with a resistor R were chosen. The CPE was used to fit the data for CNT films due to their rough and porous surface, whereas a normal capacitor was chosen to fit the data for planar platinum surfaces [35,36]. In the term for the CPE, the exponent α appears which is given in Table 4. (TIF) [file pone.0158571.s005.tif]

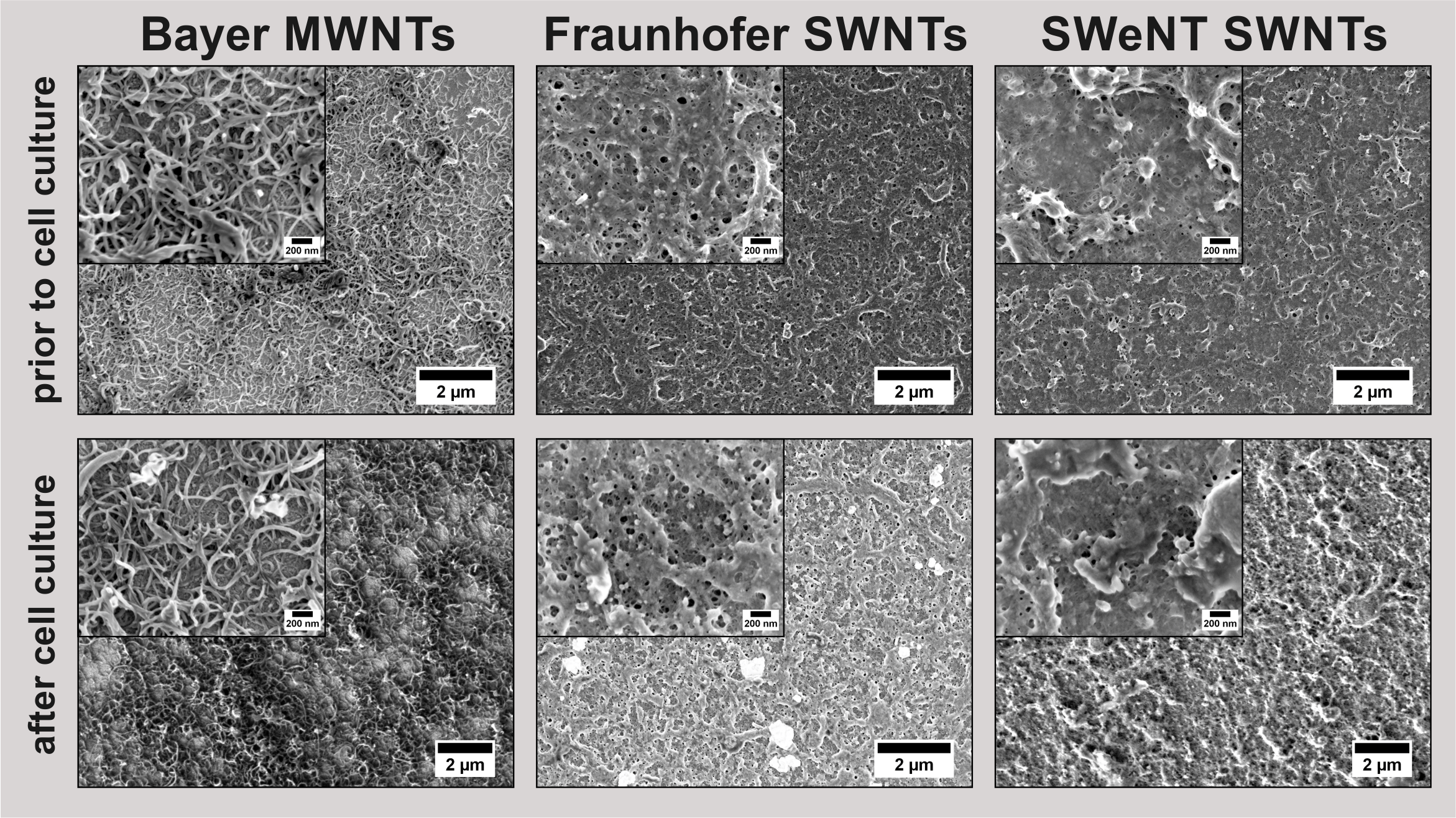

Supplement: S6 Fig — Comparison of SEM images of carbon nanotube films on platinum substrates prior and after cell culture experiments with NIH3T3 fibroblastes: Bayer MWNTs, Fraunhofer SWNTs, SWeNT SWNTs (left to right). The fibroblasts were detached via trypsin/EDTA solution after the cell culture experiments and prior to the SEM investigations. No changes of the CNT coatings are visible. (TIF) [file pone.0158571.s006.tif]

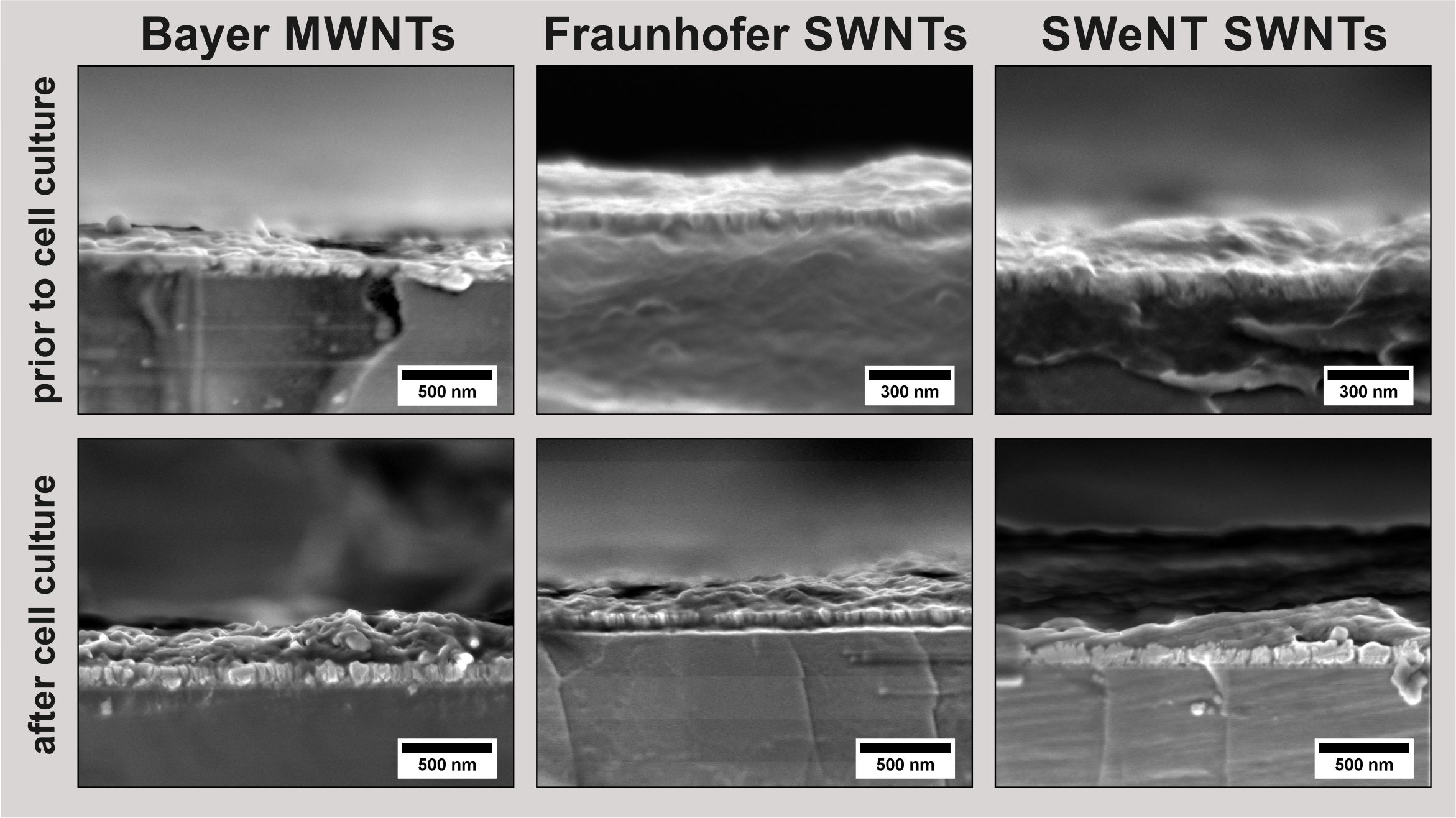

Supplement: S7 Fig — Comparison of cross-section-SEM images of carbon nanotube films on platinum substrates prior and after cell culture experiments with NIH3T3 fibroblastes: Bayer MWNTs, Fraunhofer SWNTs, SWeNT SWNTs (left to right). The fibroblasts were detached via trypsin/EDTA solution after the cell culture experiments and prior to the SEM investigations. No changes in film thickness of the CNT coatings are visible. (TIF) [file pone.0158571.s007.tif]

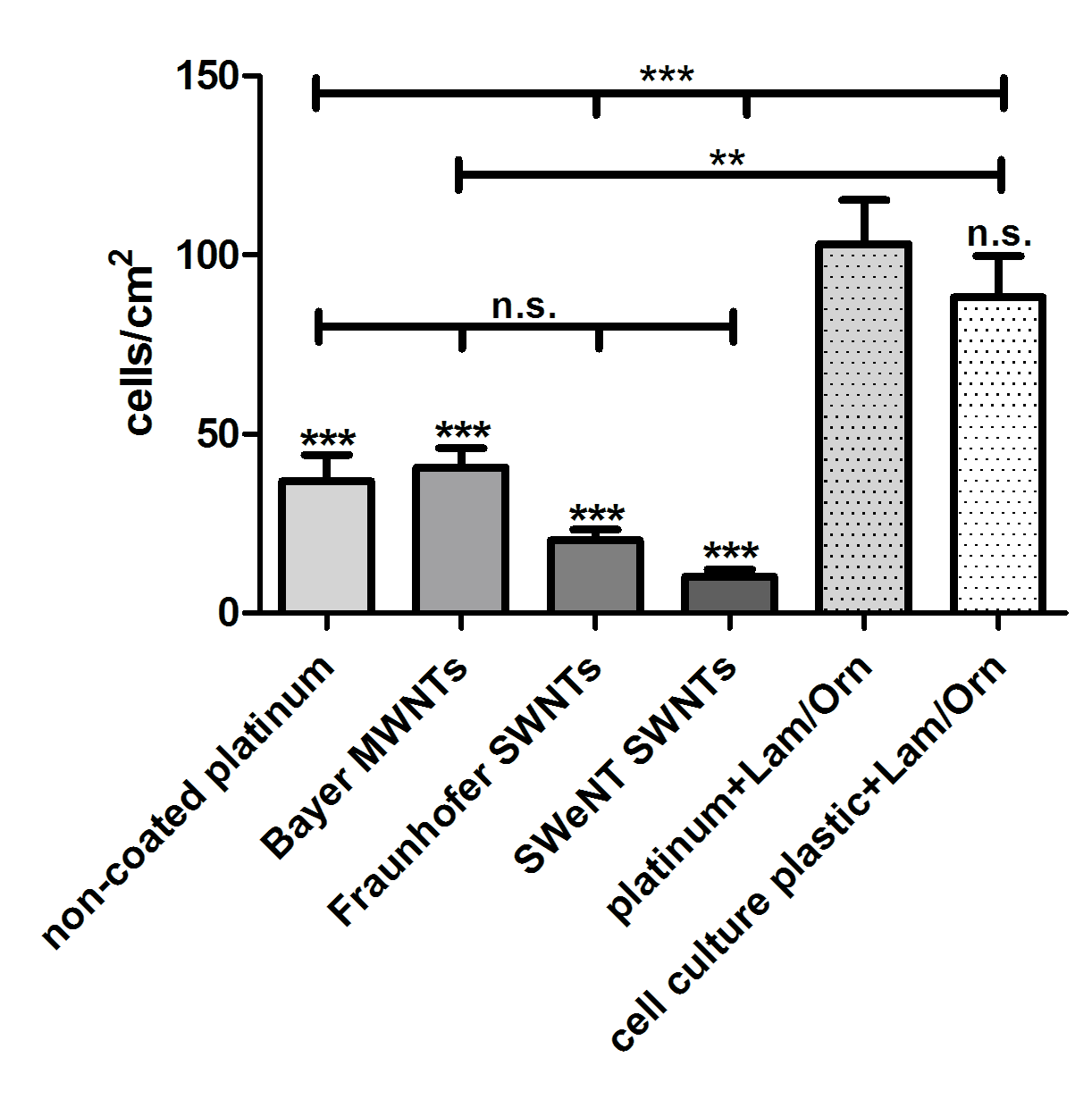

Supplement: S8 Fig — Cells were grown on CNT-coated platinum substrates and non-coated platinum as well as, for comparison, on non-coated platinum and cell culture plastic (ccp), both coated with laminin and poly-D/L-ornithine. Neurofilament-positive cells were counted under the fluorescence microscope. Values are given as mean ± standard error of the mean; N = 4, n = 2. Asterisks indicate the significance of cell per cm2 of the different samples compared to the laminin- and poly-D/L-ornithine-coated platinum. Statistical assessment was performed using one-way ANOVA with Bonferroni's multiple comparison test (n.s. = not significant; *p < 0.05; **p < 0.01; ***p < 0.001). (TIF) [file pone.0158571.s008.tif]
